# Developing prognostic models for health care utilization in patients with work-related mental health problems

**DOI:** 10.1186/s12913-023-09802-z

**Published:** 2023-08-07

**Authors:** Morten Vejs Willert, David Høyrup Christiansen, Ligaya Dalgaard, Jesper Medom Vestergaard, Johan Hviid Andersen, Marianne Kyndi

**Affiliations:** 1 Dept. of Occupational Medicine, Danish Ramazzini Centre, Aarhus University Hospital, Juul-Jensens Boulevard 35, Aarhus N, DK-8200 Denmark; 2 Research, Regional Hospital Central Jutland, Viborg, Denmark; 3 Department of Clinical Medicine, Health, Aarhus University, Aarhus, Denmark; 4 Elective Surgery Centre, Regional Hospital Silkeborg, Silkeborg, Denmark; 5 Dept of Occupational Medicine, University Research Clinic, Danish Ramazzini Center, Regional Hospital Gødstrup, Herning, Denmark; 6 Department of Psychology and Behavioral Sciences, Aarhus University, Aarhus, Denmark; 7 Danish Ramazzini Centre, Dept. of Occupational and Environmental Medicine, Aalborg University Hospital, Aalborg, Denmark

**Keywords:** Prediction model, Occupational health, Epidemiology, Stress, Occupational disease

## Abstract

**Background:**

The long-term prognosis for employees with work-related mental health problems is unclear. We aim to describe long-term trends in health care utilization (HCU) and develop multivariable prognostic models for long-term mental health care utilization.

**Methods:**

From the Danish Occupational Medicine Cohort we included mental health patients (N = 17,822) assessed from 2000 to 2013 at Departments of Occupational Medicine. Outcomes were general health (general practitioner, somatic hospital) and mental health (psychiatrist/psychologist, psychiatric hospital) HCU obtained from registries five years before/after assessment. The 10-year period was divided into phases relative to assessment: 5 − 3 years before, 2 years before/after, and 3–5 years after. We developed gender-stratified Lasso-penalized multivariable prognostic models for HCU 3–5 years after assessment assessing both calibration and discrimination.

**Results:**

Prevalent HCU for general practitioner, psychiatrist/psychologist and psychiatric hospital services was relatively stable 5 − 3 years prior to assessment, then rising during the 2 years before/after. At 3–5 years after assessment prevalent general practitioner HCU declined to previous levels, while prevalent HCU for psychologist/psychiatrist and psychiatric hospital services remained elevated compared to previous levels during years 5 − 3. Prognostic models for long-term psychologist/psychiatrist and psychiatric hospital HCU indicated acceptable calibration and modest discrimination.

**Conclusions:**

Prevalent HCU rose two years before/after assessment and remained elevated for psychiatrist/psychologist and psychiatric hospital HCU 3–5 years after. Gender-stratified prognostic models were developed for long-term mental health HCU, but discrimination and calibration should be further improved before out-of-sample application for personal prognosis.

**Trial registration:**

The study was registered at clinicaltrials.gov (Identifier: NCT04459793) prior to analyses.

**Supplementary Information:**

The online version contains supplementary material available at 10.1186/s12913-023-09802-z.

## Introduction

Patients with work-related mental health problems referred to Departments of Occupational Medicine in Denmark have increased the past 20 years, but little is known about their long-term health trends. Do their health problems represent a temporary setback or a decline towards future deteriorated health?

Previous research is scarce and we have not found any studies of long-term health care utilization for workers with work-related mental health problems. However, we did find earlier cohort studies that focus on return-to-work for workers with common mental disorders, which may prolong the return-to-work process compared to other health problems [1] and decrease worklife expectancy [2]. Also, follow-up of a clinical sample drawn from the general population indicate that patients with common mental disorders have increased use of mental health care services 6 years after baseline [3].

Danish Departments of Occupational Medicine see patients referred mainly from general practitioners (GP) and to a lesser extent (< 10%) from trade unions for assessment of the contribution from the work environment to the patient’s health problem, notification of occupational diseases, return-to-work counselling, and recommendations to the GP or the workplace regarding further initiatives. Some departments provide interventions for work-related stress which have been evaluated in randomized controlled trials [4–6], but follow-up has been limited to one year. Two studies investigated cognitive impairment in stress patients using neuropsychological testing and followed workers for up to four years, finding reduced cognitive functioning compared to healthy controls [7–9] and in one study 63% of patients felt only slightly/partly recovered after four years [10]. Thus, work-related stress may have long-term consequences, but results are based on small and selected samples. Also, patients with depression, anxiety or post-traumatic stress disorder (PTSD) are not included in the above studies. From systematic reviews of work stress interventions [11, 12] the majority of studies do not follow up beyond 12 months and thus do not illuminate the long-term health trends. To ameliorate this, we have established a patient cohort comprising all referrals to Departments of Occupational Medicine in Denmark from 2000 to 2018. In the current study, we focus on patients with work-related mental health problems between 2000 and 2013 and follow their health care utilization (HCU) for five years after their assessment by either an occupational physician or psychologist. The Danish health care system is based on the GP as the primary health care professional and covers the entire population [13]. The GP provides generalized health care and acts as a gateway to more specialized primary care, i.e. private practice psychiatrists, psychologists or physiotherapists licensed by the public health insurance, or secondary care at somatic or psychiatric hospitals. These services are covered by the public health insurance and thus free of charge to citizens, with the exception of private practice psychologist services, in which 40% of the fee is paid by the citizen. Most services are therefore available regardless of financial means, ensuring comprehensive follow-up of the endpoints in this study.

We aim to (1) describe trends of utilization across primary and secondary public health care services among patients with work-related mental health problems referred to Departments of Occupational Medicine in 2000–2013, and (2) develop prognostic models for future long-term mental health care utilization.

## Methods

Following key recommendations for developing prognostic models from Riley et al. [14] we registered the study on 07/07/2020 at clinicaltrials.gov (Identifier: NCT04459793) prior to analyses.

### Participants and source of data

We established the Danish Occupational Medicine Cohort (DOMC) [15] containing 145,390 patients assessed at Departments of Occupational Medicine in Denmark from 2000 to 2018 according to the Danish National Patient Registry (NPR) [16] and linked these to national registries and the Danish Occupational Cohort (DOC*X) [17]. As our long-term follow-up period was five years before and after the assessment and registries were complete from 1995, first year of inclusion was 2000 and last year was 2013, as 2018 was the latest available year with updated registries available. In the present study only patients diagnosed with mental health problems were included.

### Outcomes

Register data for each of five years after referral provided information on service utilization, defined as yearly summed primary and secondary care services. This included GP, private practice psychologist/psychiatrist services as well as somatic and psychiatric in-/outpatient hospital services. Registrations for GP and psychologist/psychiatrist utilization stem from The National Health Insurance Service Register [18], while somatic and psychiatric hospital services stem from the NPR. Both registers specify types of services provided, i.e. a consultation, but not the content of the consultation.

Based on trends of services across the 5 years before and after assessment, the 10-year period was split into three phases: the long-term prior phase from 5 − 3 years before assessment, the acute phase spanning two years on either side of assessment, and the long-term future phase spanning 3–5 years after assessment. For the prediction models, use of psychologist/psychiatrist and psychiatric hospital services, the outcomes were dichotomized as no/any use during years 3–5, with any use as the outcome to predict.

### Predictors

Age was calculated as the difference between date of birth and the assessment date. Marital status was divided into married, single, divorced or widowed, while further education was split into none, short (1 year), medium (3 years) or long (5 + years). Due to few patients in several occupational groups, the nine major ISCO-88 groups were reduced to five categories by merging managers and professionals, services and sales workers, and manual workers (crafts workers, agriculture/fishery/forestry, and plant/machine operators) plus armed forces. Sick leave status at assessment was retrieved from the DREAM database which contains information on long-term sick leave compensations. Departments of Occupational Medicine were grouped according to the current division of five regions within Denmark. Diagnoses given at the assessment were obtained from the NPR and grouped within 5 categories (Work-related Stress, Anxiety, Depression, PTSD and Other mental diagnoses). In daily clinical practice at the departments of occupational medicine, and also in parts of the scientific literature, it has been a tradition to place work-related stress under Adjustment disorder (F43.2) or Reactions to severe stress (F43.9). Hence, the Work-related stress category is comprised of F43.2 and F43.9, but additionally also Z-codes related to strain at work (Z56) and burn-out (Z73). The remaining groups are formed by F-codes in the following chapters: Anxiety (F40-41), Depression (F31-32), PTSD (F43.1), while Others comprises diagnoses not represented in previous categories, spanning all main chapters (F0-9) in ICD-10. Prior HCU were calculated as the cumulated number of HCU in year − 3 and split as high/low at the 75-percentile. Prior work participation was estimated as cumulated weeks fully working divided by the potential number of weeks one could work of year − 3, with > 75% defining high work participation. The Charlson Comorbidity Index was calculated from the NPR data from 1995 and up to the date of assessment, and split as none/any comorbid somatic disease.

### Sample size

No formal sample size calculation was performed as the study was based on a convenience sample. Traditionally, prognostic models require 10 outcome events per parameter in the predictors [14], equalling 350 outcome events with 35 parameters in the 12 candidate predictors.

### Missing data

Predictor variables had relatively low (0.1–2.7%) levels of missing data. We decided to include all variables in the analyses and perform complete-case analysis on all patients with no missing data on any predictor.

### Statistical analysis methods

Utilization of services and the distribution of covariates within predictors differed between men and women, and we decided to specify and present separate prognostic models for men and women. This decision was also based on suspected effect modification of gender and several demographic characteristics in relation to the HCU endpoints of the models.

Among predictors 12 were categorical, while age was kept on a continuous scale. To control for time, the year of assessment (2000–2013) was included as a continuous predictor in the models.

We built prediction models in Stata 16 (STATA Corp, College Station, Texas) using logistic regression with predictors as independent variables and long-term outcomes measured across years 3–5 after assessment as the dependent variable. From the full models with all predictors included, we applied penalized estimation using Lasso with 10-fold internal cross-validation [14].

Performance measures included Hosmer-Lemeshow Goodness-of-fit for the full models. To account for sample size, we opted for more than the usual 10 groups for the test, resulting in 40 groups for men and 120 groups for women, each holding roughly 100 patients. For the penalized models we estimated Lasso Goodness-of-fit using deviance and deviance ratio. To assess calibration, we plotted expected against observed probabilities of outcomes with added Lowess smoother. Estimates of discrimination were achieved by calculating the area under receiver operating curve (ROC AUC) and Brier score with Spiegelhalter’s z-statistic.

## Results

### Participants

A total of 17,938 patients were referred during 2000–2013 and after excluding patients under the age of 18 (n = 5), above the national retirement age of 67 years (n = 38), and those that had entered early retirement at the time of their assessment (n = 73), the study population comprised 17,822 patients with complete follow-up on the register-based outcomes. During five years of follow-up 102 patients emigrated and 205 died; these were censored on all outcomes from the year following their status change.

Demographic and health characteristics are shown in Table 1. The majority are women, comprising almost three out of four patients. On most characteristics we see differences in the distribution between men and women. The majority of patients are middle aged with a mean age of 45.6 years (SD 9.3) and more than half are married, one in four is single, and the remaining either divorced or widowed. For all patients, three out of four have short-to-medium levels of further education after primary or high school. One in five has no further education and one in twenty have long levels of further education. Men tend to be less educated than women. Occupational groups reflect educational levels as more than half of patients work as technicians and associate professionals, services and sales workers, and clerical support workers, jobs that typically require medium-to-short levels of further education. Managers/professionals and manual workers comprise the remaining occupational groups. Almost twice as many women as men work as technicians and associate professionals, services and sales workers, and clerical support workers, conversely five times as many men are manual workers or in the armed forces. The percentage of missing data on education and occupational group is low. Three out of four patients are on sick leave at initial assessment. All five Danish geographical regions are represented in the sample with the South Denmark Region and Central Denmark Region having two departments within each region, explaining their relatively higher number of patients. Work-related stress comprises almost three out of four diagnoses given, followed by depression, other mental diagnoses, PTSD and anxiety. Fewer men than women are given a diagnosis of work-related stress, while almost twice as many men are diagnosed with PTSD or within the category of other mental diagnoses. More than nine in ten have no comorbid disease.

**Table 1:** Demographic and health characteristics at the time of assessment for all patients and stratified by sex

Characteristic	All (N = 17,822)	Men (N = 4,802)	Women (N = 13,020)
Age (mean (SD))	45.6 (9.3)	46.4 (9.7)	45.4 (9.2)
Marital status			
Married	10,493 (58.9)	2,871 (59.8)	7,622 (58.5)
Single	4,267 (23.9)	1,272 (26.5)	2,995 (23.0)
Divorced	2,838 (15.9)	608 (12.7)	2,230 (17.1)
Widow	206 (1.2)	40 (0.8)	166 (1.3)
Missing	18 (0.1)	11 (0.2)	7 (0.1)
Level of further education			
None	3,172 (17.8)	1,165 (24.3)	2,007 (15.4)
Short	6,678 (37.5)	2,142 (44.6)	4,536 (34.8)
Medium	6,780 (38.0)	1,119 (23.3)	5,661 (43.5)
Long	1,062 (6.0)	313 (6.5)	749 (5.8)
Missing	130 (0.7)	63 (1.3)	67 (0.5)
Occupational groups (ISCO-88)			
Managers and professionals	3,622 (20.3)	1,010 (21.0)	2,612 (20.1)
Technicians and associate professionals	5,994 (33.6)	996 (20.7)	4,998 (38.4)
Clerical support workers	1,725 (9.7)	256 (5.3)	1,469 (11.3)
Services and sales workers	3,539 (19.9)	694 (14.5)	2,845 (21.9)
Manual workers and armed forces	2,685 (15.1)	1,715 (35.7)	970 (7.5)
Missing	257 (1.4)	131 (2.7)	126 (1.0)
On sick leave at index date			
No	4,618 (25.9)	1,371 (28.6)	3,247 (24.9)
Yes	13,204 (74.1)	3,431 (71.4)	9,773 (75.1)
Departments of Occupational Medicine,			
by geographical regions			
Capital Region of Denmark	2,094 (11.7)	711 (14.8)	1,383 (10.6)
Region of Zealand	2,176 (12.2)	575 (12.0)	1,601 (12.3)
Region of Southern Denmark	4,897 (27.5)	1,263 (26.3)	3,634 (27.9)
Central Denmark Region	5,850 (32.8)	1,492 (31.1)	4,358 (33.5)
North Denmark Region	2,805 (15.7)	761 (15.8)	2,044 (15.7)
Diagnostic groups			
Work-related stress	12,856 (72.1)	3,018 (62.8)	9,838 (75.6)
Anxiety disorder	405 (2.3)	136 (2.8)	269 (2.1)
Depression	2,284 (12.8)	671 (14.0)	1,613 (12.4)
PTSD	1,039 (5.8)	457 (9.5)	582 (4.5)
Other mental diagnosis	1,238 (6.9)	520 (10.8)	718 (5.5)
Comorbid somatic disease			
No	16,391 (92.0)	4,419 (92.0)	11,972 (92.0)
Yes	1,431 (8.0)	383 (8.0)	1,048 (8.0)

Note: Data are presented as mean (SD) for continuous measures, and n (%) for categorical measures

Abbreviations used: SD: Standard deviation, PTSD: Post-traumatic Stress Disorder

**Fig. 1:**
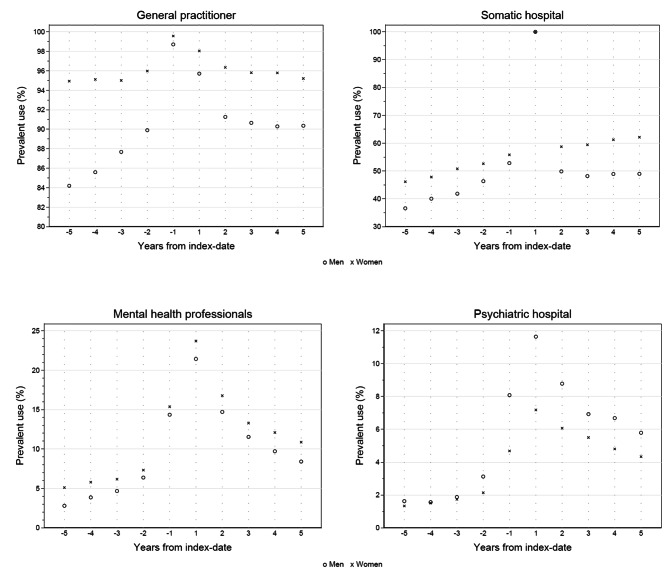
Prevalent use percentage for men (o) and women (x) across four types of health care services during +/-5 index years before and after assessment at a department of occupational medicine for a work-related mental health problem

In Table 2 prevalent, geometric mean and total utilization for each year is presented. Prevalent use refers to the percentage of the population using each service at least once per year, while the geometric mean and total use is services used among prevalent users per year. There are four types of services presented in the table (GP, somatic hospital, psychologist/psychiatrist, and psychiatric hospital), with each having a relatively stable utilization from years − 5 to -3, rising to a peak in years − 2 to 2, then gradually declining through years 3 to 5, though to a level that in years 3 to 5 is higher than at the onset in years − 5 to -3. See also Fig. 1 for a visualization of gender-stratified prevalent use percentages for the four types of health care service during 5 years before and after assessment.

**Table 2:** Health care utilization presented as prevalent use in percent, geometric mean and total services use for +/-5 index years before and after the date of assessment for four types of health care services. Results shown for all patients and stratified by sex

		Years (+/-5) before and after assessment									
Type of health care service	Metric	-5	-4	-3	-2	-1	1	2	3	4	5
General practitioner	Prevalent use (%)^a^	92	92.5	93	94.3	99.3	97.4	94.7	93.8	93.5	92.7
	Men	84.2	85.6	87.7	89.9	98.7	95.7	91.3	90.7	90.3	90.4
	Women	94.9	95.1	95	96	99.6	98	96.3	95.8	95.8	95.2
	Geometric mean^b^	6	6.1	6.5	6.9	10.2	8.8	7.5	7.4	7.3	7.4
	Men	4.4	4.6	5	5.7	8.8	7.6	6.3	6.4	6.3	6.3
	Women	6.6	6.8	7.1	7.4	10.8	9.3	7.9	7.8	7.8	7.8
	Total prevalent use^b^	138,824	143,524	151,050	162,311	227,796	204,327	176,979	174,293	172,135	171,481
Somatic hospital	Prevalent use (%)^a^	43.6	45.7	48.4	50.9	55	100	56.2	56.1	57.4	57.9
	Men	36.6	40	41.8	46.3	52.8	100	49.8	48.2	48.9	49
	Women	46.1	47.8	50.8	52.6	55.8	100	58.7	59.4	61.2	62.1
	Geometric mean^b^	3.2	3.2	3.3	3.3	3.5	5	3.8	3.8	3.9	3.9
	Men	2.9	2.9	3.1	3.2	3.8	4.6	3.7	3.7	3.9	3.9
	Women	3.3	3.4	3.4	3.4	3.5	5.1	3.8	3.9	3.9	3.9
	Total prevalent use^b^	36,599	38,499	42,413	44,107	50,366	118,833	58,477	58,362	61,621	61,214
Psychologist/psychiatrist	Prevalent use (%)^a^	4.5	5.3	5.8	7.1	15.1	23.1	16.2	12.7	11.3	10.1
	Men	2.8	3.9	4.7	6.4	14.3	21.4	14.7	11.5	9.7	8.4
	Women	5.1	5.8	6.2	7.3	15.4	23.7	16.8	13.3	12.1	10.9
	Geometric mean^b^	3.8	3.8	4.1	4.2	4.5	5.4	4.9	4.7	4.6	4.5
	Men	3	3.6	3.8	4	4.3	5.2	4.9	4.6	4.5	4.1
	Women	3.9	3.8	4.1	4.3	4.6	5.5	4.9	4.7	4.7	4.6
	Total prevalent use^b^	4,022	4,809	5,667	7,237	16,552	30,629	19,817	14,718	12,970	11,031
Psychiatric hospital	Prevalent use (%)^a^	1.4	1.5	1.8	2.4	5.6	8.4	6.8	5.8	5.3	4.7
	Men	1.6	1.6	1.9	3.1	8.1	11.6	8.8	6.9	6.7	5.8
	Women	1.3	1.5	1.7	2.1	4.7	7.2	6.1	5.5	4.8	4.3
	Geometric mean^b^	5.3	4.9	4.5	4.8	4.7	6.8	7.2	6.8	6.5	7.7
	Men	3.2	4.1	3.7	4.4	4.4	6.6	6.8	6.8	5.6	6.8
	Women	6.6	5.2	4.9	4.9	4.9	7	7.5	6.8	7.1	8.1
	Total prevalent use^b^	2,650	2,518	2,984	3,840	8,744	18,214	15,684	13,329	11,045	11,277

Notes: ^a^ Prevalent use refers to the percentage of the population using each service at least once per year. ^b^ Geometric mean and total use are based on services used among prevalent users per year

For GP services Table 2 shows 92.0% prevalent utilization in year − 5, rising to 99.3% (year − 1), then declining to 92.7 (year 5), though generally lower for men than women (see also Fig. 1). The geometric mean utilization starts at 6.0 in year − 5 rising to 10.2 (year − 1), then declining to 7.3–7.4 (years 3–5), generally higher for women than men.

Somatic hospital prevalent utilization rises incrementally from 43.6% (year − 5) to 57.9% (year 5), with the exception of year 1 at 100% due to their contact to a Department of Occupational Medicine. The incidence is generally lower for men than women (see also Fig. 1).

Prevalent psychologist/psychiatrist utilization is 4.5% in year − 5, rising to 23.1% (year 1), then declining to 10.1% (year 5), with incidence for women above men, though the gap narrows over time (see also Fig. 1). Prevalent and total utilization more than double comparing year − 5 to 5.

For psychiatric hospital services prevalent utilization is 1.4% in year − 5, peaking at 8.4% (year 1), then declining to 4.7% (year 5). Contrary to the above, men have the larger use percentage, (see also Fig. 1). Prevalent and total utilization more than triple comparing year − 5 to 5.

### Model development

The prognostic models were developed using logistic regression with dichotomized outcomes for use of psychologist/psychiatrist and psychiatrist hospital services, both of which had the most prominent change from the prior phase 5 − 3 years before assessment to the long-term future phase 3–5 years after assessment. Predictors included in the full models are age, marital status, level of further education, major occupational group, geographical regions, diagnostic groups, sick leave status at assessment, prior use of psychologist/psychiatrist and psychiatric hospital services, prior work participation, comorbid somatic disease, and calendar year. Of the study population described in Table 1, 5.2% men and 2.0% women were excluded due to missing data on one or more of these predictors.

Models were developed separately for men (N = 4,554 complete cases) and women (N = 12,757 complete cases). Analyses for men are most critical in terms of power, with 796 outcome events for psychologist/psychiatrist and 530 outcome events for psychiatric hospital, both above the 350 required outcome events outlined earlier. In models developed for 12,757 women psychologist/psychiatrist and psychiatric hospital services hold 2,685 and 1,127 outcome events.

### Model specification

In Table 3 results for the prognostic models for men and women are presented. In the first column for men, the full psychologist/psychiatrist model predicts that individuals belonging to the base category [1] on all included risk factors have odds 0.51 for using any such services during years 3–5 after assessment. When calculating individual prognoses, the model specifies that the base odds should be multiplied by the odds ratio for each of the non-base categories the individual belongs to. So for example a single male sales worker with depression and prior high use of psychologist psychiatrist services would have the base odds 0.51 multiplied by 0.95 (divorced OR), 0.99 (Services/sales workers OR), 1.68 (depression OR), and 2.83 (prior use OR) resulting in predicted odds of 2.28 from the full model.

**Table 3:** Multivariable prognostic models of risk factors for health care utilization (HCU) during years 3–5 after assessment among mental health patients referred during 2000–2013 to Departments of Occupational Medicine in Denmark. Separate models for men and women are presented for each of four types of HCU, with risk estimates expressed as odds ratios (OR) for both full models and penalized Lasso models. Private practice psychologists/psychiatrists and psychiatric hospital in-/outpatient HCU are dichotomized as prevalent use (none vs. any) across years 3–5

		Men (N = 4,554)				Women (N = 12,757)			
		Psychologist/ psychiatrist		Psychiatric hospital		Psychologist/ psychiatrist		Psychiatric hospital	
Risk factor	Range/categories	Full	Lasso^a^	Full	Lasso^a^	Full	Lasso^a^	Full	Lasso^a^
Age	18–67 years	0.99	0.99	0.97	0.98	0.99	0.99	0.97	0.98
Marital status	Married	1		1	0.93	1	0.8	1	0.84
	Single	1.09	1.06	1.1		1.28	1.01	1.2	
	Divorced	0.95		1.13		1.27		1.44	1.18
	Widow	0.99		1.64		0.8	0.67	0.97	0.89
Further education	Long	1	1.31	1		1	1.09	1	0.85
	Medium	0.82	1.09	0.7	0.91	0.98	1.09	1.23	
	Short	0.68	0.98	0.81		0.89		1.21	
	None	0.7		0.82		0.86	0.97	1.43	1.16
Occupational groups (ISCO-88)	Managers/professionals	1	1.08	1	1	1	1.04	1	0.85
	Technicians/assoc. professionals	0.89		1.19		0.96		1.18	
	Clerical support workers	0.74	0.9	1.06		0.97		1.21	
	Services/sales workers	0.99	1.07	1.03		0.87	0.91	1.21	
	Manual workers/armed forces	0.68	0.77	1.16		0.78	0.83	1.35	1.1
Geographical regions	Capital region of Denmark	1	1.57	1		1	1.21	1	1.26
	Region of Zealand	0.7	1.1	0.98		0.84	1.01	0.78	
	Region of Southern Denmark	0.62		1.11	1.09	0.82		0.79	1.01
	Central Denmark Region	0.51	0.87	0.76	0.86	0.65	0.8	0.68	0.89
	North Denmark Region	0.56	0.97	0.87		0.79	0.98	0.7	0.92
Diagnostic groups	Work-related stress	1	0.88	1	0.67	1	0.62	1	0.62
	Anxiety	1.15		1.13	0.95	1.76	1.06	2.5	1.5
	Depression	1.68	1.4	1.78	1.1	1.63		2.12	1.3
	PTSD	1.74	1.44	2.01	1.15	1.77	1.07	1.66	
	Other mental disorder	1.14		1.51		0.99	0.63	1.37	0.87
On sick leave at assessment	No	1	0.7	1	0.75	1	0.84	1	0.72
	Yes	1.5		1.46		1.21		1.41	
Prior psychologist/ psychiatrist use	Low	1	0.36	1	0.75	1	0.47	1	0.72
	High	2.83		1.41		2.13		1.4	
Prior psychiatric hospital use	Low	1	0.94	1	0.31	1	0.81	1	0.28
	High	1.15		3.48		1.26		3.6	
Prior work participation	High	1	0.94	1	0.91	1	0.84	1	0.8
	Low	1.12		1.15		1.21		1.26	
Comorbid somatic disease	No	1	1.08	1		1	0.85	1	0.95
	Yes	0.85		1.06		1.2		1.09	
Year of assessment	2000–2013	1.01	1	1.06	1.04	1.02	1.01	1.05	1.04

^a^ Penalizing full models using Lasso reduces the number of covariates to those essential for prognosis and shrinks effect estimates to reduce overfitting

Penalizing full models using Lasso reduces the number of covariates to those essential for prognosis and shrinks effect estimates to reduce overfitting. For men, the number of covariates in the penalized models was reduced by a third or more, from 35 in the full model to the 15–18 covariates seen in Table 3 under the LASSO columns for men regarding psychologist/psychiatrist and psychiatric hospital use. Similarly, for women the number was reduced by a third to the 22–24 covariates seen in the LASSO columns for women in Table 3. However, the more parsimonious Lasso models could sacrifice predictive performance compared to full models; hence we employed comprehensive comparison of performance measures for each type of model, as detailed below.

### Model performance

In Table 4 performance measures of the full and Lasso models are presented and compared in terms of both calibration and discrimination.

**Table 4:** Performance measures for multivariable prognostic models of risk factors for health care utilization (HCU) during years 3–5 after assessment among mental health patients referred during 2000–2013 to Departments of Occupational Medicine in Denmark. Separate models were developed for men and women and measures of calibration and discrimination are presented for psychologist/psychiatrist and psychiatric hospital HCU.

Performance measure	Men						Women					
	Psychologist/psychiatrist			Psychiatric hospital			Psychologist/psychiatrist			Psychiatric hospital		
	Full	Lasso	p	Full	Lasso	p	Full	Lasso	p	Full	Lasso	p
Calibration												
H-L G-o-f Chi2	44.1		0.23	44.6		0.21	142.93		0.06	109.03		0.71
Deviance	0.82	0.86		0.63	0.66		0.97	0.99		0.54	0.55	
Deviance ratio	0.09	0.05		0.1	0.05		0.05	0.04		0.08	0.06	
Discrimination												
ROC AUC	0.66	0.66	0.49	0.68	0.68	0.42	0.63	0.63	0.23	0.69	0.69	0.67
AUC 95% CI	0.64–0.68	0.64–0.68		0.65–0.70	0.65–0.70		0.62–0.65	0.62–0.65		0.67–0.70	0.67–0.70	
Brier score	0.13	0.13		0.09	0.09		0.16	0.16		0.08	0.08	
Speigelhalter’s z	0.46	0.62		0.45	0.61		0.45	0.55		0.4	0.46	

Abbreviations used in table: H-L: Hosmer Lemeshow, G-o-f: Goodness of fit, ROC: Receiver operating curve, AUC: Area under curve, 95% CI: 95% confidence intervals, p: p-value

For men, the full models for psychologist/psychiatrist and psychiatric hospital use had acceptable calibration regarding the Hosmer-Lemeshow Chi^2^ Goodness-of-fit with p-values > 0.05 (range: 0.21–0.23). The Lasso Goodness-of-fit deviance and deviance ratios were comparable between the full and Lasso models. Regarding discrimination, the AUC of the ROC was 0.66–0.68 with slim confidence intervals. Testing the difference between the ROC AUC of full and Lasso models we found p = 0.42–0.49, indicating that Lasso models performed equal to full models. The Brier score was 0.09–0.13 with identical scores from full and Lasso models and Speigelhalter’s z > 0.05 (range: 0.45–0.62), indicating acceptable discrimination.

For women, the full models for psychologist/psychiatrist and psychiatric hospital use had Hosmer-Lemeshow Chi^2^ Goodness-of-fit p-values > 0.05 (range: 0.06–0.71). The Lasso Goodness-of-fit deviance and deviance ratios were almost identical between the full and Lasso models. In terms of discrimination, the AUC of the ROC ranged from 0.63 to 0.69 with slim confidence intervals. Testing the difference between ROC AUC of full and Lasso models (p = 0.23–0.67) indicated that Lasso models performed equal to full models. Brier scores ranged from 0.08 to 0.16, with identical scores from full and Lasso models and Speigelhalter’s z-scores > 0.05 (range: 0.40–0.55) indicate acceptable discrimination.

ROC-curves for Lasso models of psychologist/psychiatrist and psychiatric hospital use are presented in Fig. 2 (see supplementary materials) for both men and women.

In Fig. 3 (see supplementary materials) we present calibration plots of the Lasso models for men and women for both psychologist/psychiatrist and psychiatric hospital use. In the plots, patients are grouped by increasing predicted risk of the outcome. Models for both men and women discriminate poorly between groups of patients with lower risk, while the group with the highest risk is separated from the remaining nine groups with a lower risk.

## Discussion

In a cohort of 17,822 patients assessed at Departments of Occupational Medicine during 2000–2013 for work-related mental health problems, we examined four types of HCU for 5 years before and after assessment. For GP, psychologist/psychiatrist and psychiatric hospital services, we observe a pattern of stable utilization 3–5 years prior to assessment, rising to a peak during the two years on either side of assessment, declining to a new level 3–5 years after, though above that of 3–5 years prior to assessment. Prevalent use of somatic hospital services exhibits a steady rise from 5 years prior to 5 years after assessment, with the exception of year 1 in which all patients have a somatic hospital contact due to the inclusion criteria of visiting a Dept. of Occupational Medicine.

For the two mental health outcomes we developed prognostic models for both men and women and successfully applied penalized estimations using Lasso, reducing the number of covariates by approximately a third and shrinking predictor effect estimates to reduce overfitting when potentially transferring the model to future out-of-sample patients. Performance measures of the models demonstrated acceptable calibration and modest discrimination, though calibration plots indicated better performance for the 10% of patients with the highest risk of psychologist/psychiatrist or psychiatric hospital use.

### Strengths and limitations

It is a strength that we have a large, nation-wide study population, with follow-up spanning five years before and after assessment and demographic, work and health-related predictor variables with few missing data. Complete follow-up of outcomes strengthens the internal validity of the results and statistical power.

However, the study has a number of limitations that may threaten both the internal and external validity. We lack information about life style factors such as smoking, alcohol consumption, dietary habits, physical exercise and general fitness that are known to impact HCU. Also, we have no data on new-onset somatic disease or traumatic life events during follow-up after assessment, and have no information on genetic mental health dispositions.

Regarding external validity, we lack a matched control group to compare HCU in all phases of the 10-year follow-up period. Furthermore, even though the Danish healthcare system is free and accessible to all citizens, for psychologist/psychiatrist services we lack information for services provided outside of The National Health Insurance Service Register. This is especially true for services by private practice psychologists, as only treatment for mild-moderate depression and anxiety is covered. Patients with work-related stress are thus not covered and will either pay full price or use a private health insurance or pension policy. The latter has been on the rise in Denmark during the inclusion period, especially in the private job sector but no central registrations of such services exist. This could lead to an underestimation of psychologist/psychiatrist HCU for patients with work-related stress.

A potential difficulty in the analyses was how HCU, which approximates a negative binomial distribution, should be handled. As Lasso procedures are not available for negative binomial regression models, we decided to dichotomize the outcomes allowing for logistic regression analyses. For the mental health outcomes (psychologist/psychiatrist and psychiatric hospital services) we decided to dichotomize on none vs. any HCU, as the primary concern is not how many consultations the patient has used, but if they have crossed the threshold of referral to mental health care.

## Conclusions

Though we were successful in specifying penalized models with performance measures equal to the full models and hence reduce the risk for over-optimism when applied to future out-of-sample patients, the ROC AUC estimates across all models of 0.63–0.69 are barely sufficient for population level prognosis and below preferred thresholds for personalized prognosis.

In a daily clinical setting, it is a common concern for both patients and clinicians, if the presented mental health problem will be not only a temporary setback, but a decline towards more long-term and chronic health problems. We see a rise in psychologist/psychiatrist and psychiatric hospital services in the two years on either side of assessment, but in the long-term future phase during years 3–5, most patients do not receive these mental health-specific services, with 9 out of 10 patients not using psychologist/psychiatrist services and 19 out of 20 not using psychiatric hospital services.

Next steps to improve the prognostic models are to obtain data on life style and symptom severity from patient records, traumatic life events (death or disease in the family, divorce) and mental health problems among parents or siblings. We plan to include a matched control group in the next update of the Danish Occupational Medicine Cohort and update data beyond the current end-of-follow-up in 2018.

### Electronic supplementary material

Below is the link to the electronic supplementary material.

Supplementary Material 1

Supplementary Material 2

## Data Availability

Access to data is obtained through the cohort executive committee at the Regional Hospital West Jutland with a short project description. Further information on content and variables of the Danish Occupational Medicine Cohort and how to gain access is available through personal contact of the chairman of the executive committee by mail morten.willert@aarhus.rm.dk. Individual-level data in the database is available only through online access at Statistics Denmark under standard conditions.

## List of abbreviations

HCU: Health care utilization
GP: General practitioner
NPR: National Patient Register
ROC: Receiver-operator-curve
AUC: Area-under-curve
