# Supplementary material for: Developing prognostic models for health care utilization in patients with work-related mental health problems
Source: BMC Health Serv Res. 2023 Aug 7;23:834. doi: 10.1186/s12913-023-09802-z (PMC10405445; doi:10.1186/s12913-023-09802-z)
Supplement: Supplementary file 1 — Supplementary Material 1 [file 12913_2023_9802_MOESM1_ESM.docx]

**Figure 2.** ROC-curves for multivariable prognostic models for four types of heath care utilization. Models are developed for men and women separately and penalized using Lasso to reduce the number of covariates and restrict over-optimism in the models.
