# Supplementary material for: Developing prognostic models for health care utilization in patients with work-related mental health problems
Source: BMC Health Serv Res. 2023 Aug 7;23:834. doi: 10.1186/s12913-023-09802-z (PMC10405445; doi:10.1186/s12913-023-09802-z)

**Figure 3.** Calibration plots of multivariable prognostic models for four types of heath care utilization. Models are developed for men and women separately and penalized using Lasso to reduce the number of covariates and restrict over-optimism in the models. In the plots the study sample is divided into 10 groups of rising predicted probability of the outcome, and for each group is shown the expected against observed probabilities of using psychologist/psychiatrist or psychiatric hospital services, with accompanying confidence intervals and the Lowess smoother. The dotted reference line delineates perfect calibration.

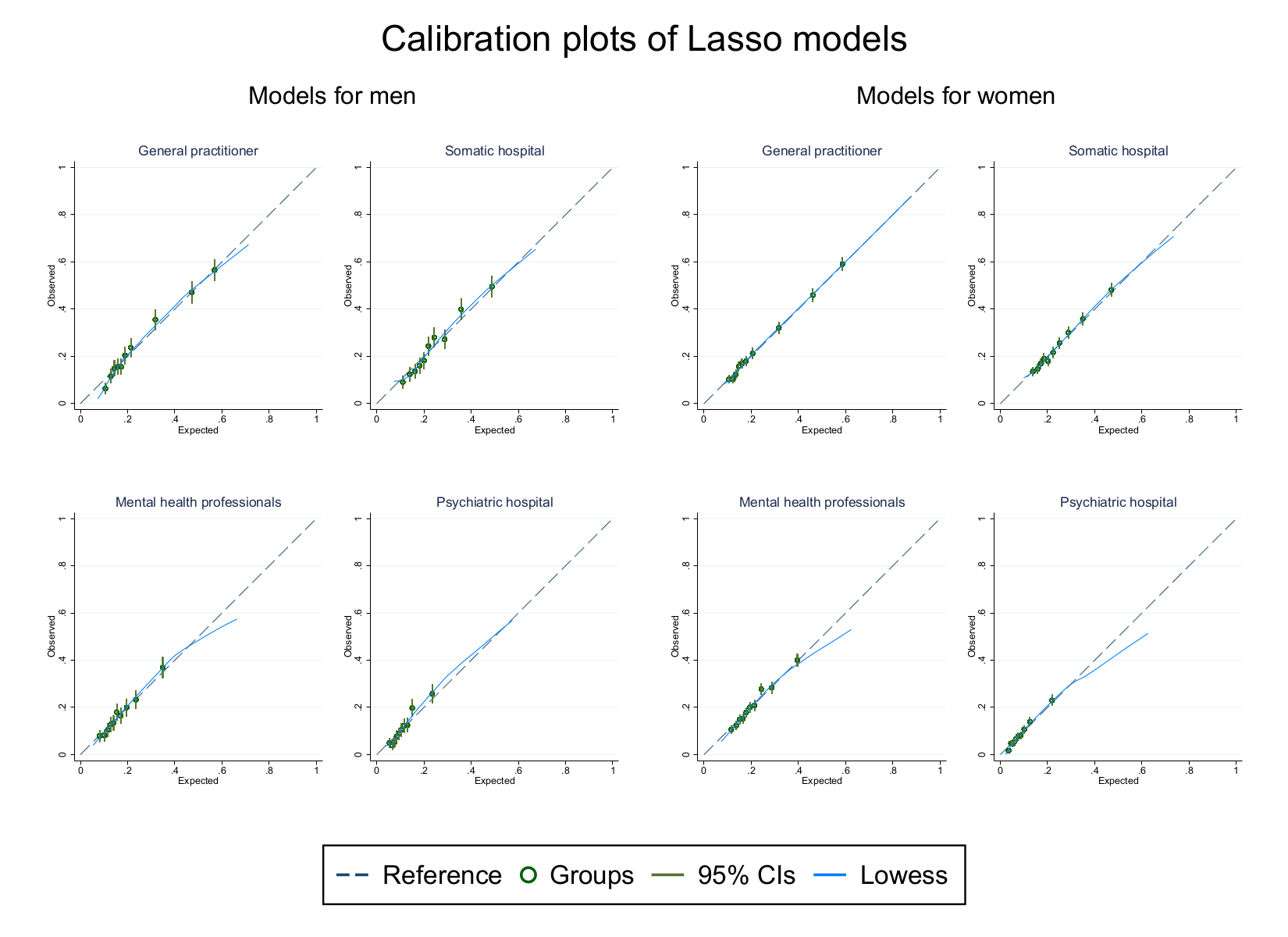

Supplement: Supplementary file 2 — Supplementary Material 2 [file 12913_2023_9802_MOESM2_ESM.docx]
